# *In Silico* Analysis for Transcription Factors With Zn(II)_2_C_6_ Binuclear Cluster DNA-Binding Domains in *Candida albicans*

**DOI:** 10.1002/cfg.492

**Published:** 2005

**Authors:** Sergi Maicas, Inmaculada Moreno, Almudena Nieto, Micaela Gómez, Rafael Sentandreu, Eulogio Valentín

**Affiliations:** 1 Departamento de Bioquímica y Biología Molecular Universitat de València Burjassot Valencia 46100 Spain; 2 Departamento de Microbiología y Ecología Facultad de Farmacia Universitat de València Avda. Vicent Andrés i Estellés s/n Burjassot Valencia 46100 Spain

## Abstract

A total of 6047 open reading frames in the *Candida albicans* genome were screened for Zn(II)_2_C_6_-type zinc cluster proteins (or binuclear cluster proteins) involved in DNA recognition. These fungal proteins are transcription regulators of genes involved in a wide range of cellular processes, including metabolism of different compounds such as sugars or amino acids, as well as multi-drug resistance, control of meiosis, cell wall architecture, etc. The selection criteria used in the sequence analysis were the presence of the CysX_2_CysX_6_CysX_5-16_CysX_2_CysX_6-8_Cys motif and a putative nuclear localization signal. Using this approach, 70 putative Zn(II)_2_C_6_ transcription factors have been found in the genome of *C. albicans*.


**Comparative and Functional Genomics**
*Comp Funct Genom* 2005; **6**: 345–356.
Published online 21 October 2005 in Wiley InterScience (www.interscience.wiley.com). **DOI:** 10.1002/cfg.492

**Research Article**

### *In silico* analysis for transcription factors with $Zn(II)_2C_6$ binuclear cluster DNA-binding domains in *Candida albicans*

Sergi Maicas[1], Inmaculada Moreno[2], Almudena Nieto[2], Micaela Gómez[1], Rafael Sentandreu[2] and Eulogio Valentín[2]*

[1]*Departamento de Bioquímica y Biología Molecular, Universitat de València, 46100-Burjassot, Valencia, Spain*
[2]*Departamento de Microbiología y Ecología, Universitat de València, 46100-Burjassot, Valencia, Spain*

**Correspondence to:*
*Eulogio Valentín, Departamento de Microbiología y Ecología, Facultad de Farmacia, Universitat de València, Avda. Vicent Andrés i Estellés s/n, 46100-Burjassot, Valencia, Spain.*
*E-mail: Eulogio.Valentin@uv.es*

Received: 3 March 2005
Revised: 26 May 2005
Accepted: 5 September 2005

### Abstract

**A total of 6047 open reading frames in the *Candida albicans* genome were screened for $Zn(II)_2C_6$-type zinc cluster proteins (or binuclear cluster proteins) involved in DNA recognition. These fungal proteins are transcription regulators of genes involved in a wide range of cellular processes, including metabolism of different compounds such as sugars or amino acids, as well as multi-drug resistance, control of meiosis, cell wall architecture, etc. The selection criteria used in the sequence analysis were the presence of the $CysX_2CysX_6CysX_{5-16}CysX_2CysX_{6-8}Cys$ motif and a putative nuclear localization signal. Using this approach, 70 putative $Zn(II)_2C_6$ transcription factors have been found in the genome of *C. albicans*. Copyright © 2006 John Wiley & Sons, Ltd.**

**Keywords:** ***Candida albicans*; transcription factor; $Zn(II)_2C_6$ domain; binuclear cluster proteins**

### Introduction

Biological systems contain an important group of proteins characterized by their ability for DNA binding and participation in important processes, such as DNA replication and repair and transcription gene control. Gene expression can be controlled at various levels, including transcription, mRNA splicing, mRNA stability, translation and even post-translation events, such as protein stability and modification. There are many regulatory sequences in genes that bind various transcription factors. These regulatory sequences are essentially located upstream (5′) of the transcription initiation site, although some elements occur downstream (3′) or even within the genes themselves. The number and type of regulatory elements are variable for each gene. Moreover, various cell types express characteristic combinations of transcription factors; this is the major mechanism for cell-type specificity in the regulation of gene expression.

Transcription factors have been grouped in different families as a function of their DNA binding domains. These DNA binding domains comprise the fungal helix–loop–helix (HLH), helix–turn–helix (HTH), high mobility group (HMG) box, basic region–leucine zipper (bZIP), MADS box, TEA/ATTS domain, the zinc(II) coordinating $Cys_2His_2$, $Cys_2X_{17}Cys_2$ (GATA), copper DNA binding, heat shock transcription factor (HSTF) and $Zn(II)_2Cys_6$ binuclear cluster (Klug and Rhodes, 1987; Davis and Hynes 1987; Furst *et al.*, 1988; Nehlin and Ronne, 1990; Burglin, 1991; Jakobsen and Pelham, 1991; Todd and Andrianopoulos, 1997; Kohler *et al.*, 2002).

In fungi such as *Saccharomyces cerevisiae* (Pan and Coleman 1990; Akache *et al.*, 2001), *Aspergillus flavus* (Woloshuk *et al.*, 1994), *A. nidulans* (Ascone *et al.*, 1997), *Fusarium solani* (Li and Kolattukudy, 1997), *A. niger* (Todd *et al.*, 1997), *Kluyveromyces lactis* (Breunig and Kuger, 1987), *Neurospora crassa* (Yuan *et al.*, 1991) and

Copyright © 2006 John Wiley & Sons, Ltd.

*Schizosaccharomyces pombe* (Tang *et al.*, 1994), an important set of transcription factors is composed by a sub-family of zinc finger proteins named zinc cluster proteins or binuclear cluster proteins, $Zn(II)_2C_6$, characterized by containing the well-conserved motif $CysX_2CysX_6CysX_{5-16}CysX_2CysX_{6-8}Cys$ (Figure 1), with cystein residues binding to two zinc atoms which coordinate folding of the domain (Vallee *et al.*, 1991). In *C. albicans* only four $Zn(II)_2C_6$ proteins have been reported (Kelly and Kwon-Chung, 1992; Whiteway *et al.*, 1992; Talibi and Raymond, 1999, Moreno *et al.*, 2003). In the present study, we have taken a sequence-dependent approach to identify new $Zn(II)_2C_6$ ORFs by screening the genome database of *C. albicans* for $Zn(II)_2C_6$ transcription factors by an *in silico* analysis.

### Materials and methods

The BLAST utility provided by the *C. albicans* genome database (**http://www.pasteur.fr/recherche/unites/GalarFungail**; Altschul *et al.*, 1997) was used to search for putative transcription factors containing the $Zn(II)_2C_6$ binuclear motif. The *C. albicans* putative $Zn(II)_2C_6$ proteins were aligned using the ClustalW online interface (**http://www.ebi.ac.uk/clustalw**; Thompson *et al.*, 1994) and manual alignment. After the alignment, the output data were submitted to the Phylip drawtree web interface utility at the Institute Pasteur (**http://bioweb.pasteur.fr/seqanal/interfaces/drawgram.html**; Lim and Zhang, 1999) to get the phenogram. Comparative analysis between *S. cerevisiae* and *C. albicans* $Zn(II)_2C_6$ putative transcription factors was carried out by reciprocal analysis of the SGD (**http://www.yeastgenome.org**) and *C. albicans* database entries. SCANPROSITE (**http://www.expasy.org/tools/scanprosite**; Gattiker *et al.*, 2002) was also used for proteins matching the consensus sequence. PSORTII (**http://psort.ims.u-tokyo.ac.jp**; Horton and Nakai, 1997) was used for subcellular localization prediction. The potential of dimerization via $Zn(II)_2C_6$ structures was investigated using the COILS program (**http://www.ch.embnet.org/software/COILS_form.html**; Lupas *et al.*, 1991) as described by Taylor and Zhulin (1999).

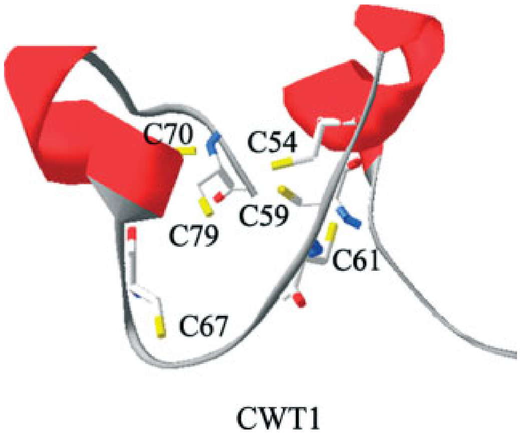

CWT1

**Figure 1.** Schematic representation of a characteristic *C. albicans* $Zn(II)_2C_6$ domain. The structure was produced by threading it to the *S. cerevisiae* Cyp1 (Hap1) DNA binding domain (PDB: 1PYC) by using the JIGSAW utility (**http://www.bmm.icnet.uk/servers/3djigsaw**; Bates and Sternberg, 1999). The position of the six cysteines is annotated. The figure was generated using the Swiss Pdb-viewer (Guex and Peitsch, 1997)

### Results and discussion

### *In silico* screening for potential $Zn(II)_2C_6$ transcription factors

The determination of the complete genomic sequence of *Candida albicans* (**http://www-sequence.standford.edu/group/candida**), annotated by the European Consortium Galar Fungail (**http://www.pasteur.fr/recherche/unites/GalarFungail**), has allowed us to search for new putative transcription factors containing the $Zn(II)_2C_6$ binuclear motif. The criterion used for selection was the presence of the $CysX_2CysX_6CysX_{5-16}CysX_2CysX_{6-8}Cys$ cysteine pattern. All the 6047 *C. albicans* ORFs were screened, based on this criterion, and a set of 70 potential $Zn(II)_2C_6$ transcription factors, including the four previously known $Zn(II)_2C_6$ proteins, viz. CaFcr1p, CaSuc1p, CaCzf1p and CaCwt1p, was generated (Table 1). In the complete genome of *S. cerevisiae* a total of 58 $Zn(II)_2C_6$ proteins have been reported (Akache *et al.*, 2001).

### Structure of the $Zn(II)_2C_6$ domain

The characteristic DNA binding domain of $Zn(II)_2C_6$ proteins contains a highly-conserved $CysX_2CysX_6CysX_{5-16}CysX_2CysX_{6-8}Cys$ motif, which was first described in *S. cerevisiae* (Pan and Coleman, 1990). In this motif the six cysteine residues

Copyright © 2006 John Wiley & Sons, Ltd. 

**Table 1.** Alternative names for $Zn(II)_2Cys_6$ proteins used in Candida DB (*C. albicans* DataBase at **http://genolist.pasteur.fr/CandidaDB**), SGTC (Stanford Genome Technology Center at **http://www-sequence.sandford.edu**) and CYGD (Comprehensive Yeast Genome Database at **http://pedant.gsf.de**)

| Candida DB | SGTC | CYGD |
|---|---|---|
| IPF100.3 | orf19.5940 | CA6113 |
| IPF376 | orf19.7518 | CA5860 |
| IPF776 | orf19.5338 | CA5497 |
| IPF907 | orf19.7583 | CA5985 |
| IPF928 | orf19.7570 | CA5976 |
| IPF1034 | orf19.4573 | CA1083 |
| IPF1040 | orf19.4568 | CA1544 |
| IPF1196 | orf19.2077 | CA4820 |
| IPF1264 | orf19.7371 | CA5669 |
| IPF1266 | orf19.7372 | CA5670 |
| IPF1292 | orf19.7381 | CA5678 |
| IPF1457 | orf19.6038 | CA4901 |
| IPF1960.5f | orf19.7318 | CA5554 |
| IPF2029 | orf19.5251 | CA4996 |
| IPF2319 | orf19.6680 | CA4282 |
| IPF3444 | orf19.6182 | CA3201 |
| IPF3781(CWT1) | orf19.5849 | CA2880 |
| IPF4835 | orf19.5992 | CA6071 |
| IPF6159 | orf19.1035 | CA1038 |
| IPF6203 | orf19.4166 | CA3716 |
| IPF6510 | orf19.1685 | CA2306 |
| IPF6554 | orf19.4450 | CA4663 |
| IPF6874.3f | orf19.4251 | CA2184 |
| IPF7221 | orf19.4046 | CA2491 |
| IPF7289 | orf19.391 | CA3878 |
| IPF7629 | orf19.1168 | CA1786 |
| IPF7952 | orf19.3305 | CA4614 |
| IPF8224 | orf19.5380 | CA2298 |
| IPF9188 | orf19.3187 | CA2539 |
| IPF9251 | orf19.5133 | CA3639 |
| IPF9312 | orf19.4649 | CA1718 |
| IPF9499 | orf19.2808 | CA2621 |
| IPF9826 | orf19.4145 | CA3088 |
| IPF10079 | orf19.2280 | CA0257 |
| IPF10197 | orf19.2753 | CA1892 |
| IPF10533 | orf19.1255 | CA3454 |
| IPF11777 | orf19.4778 | CA0777 |
| IPF13021 | orf19.2647 | CA1726 |
| IPF13024 | orf19.2646 | CA2064 |
| IPF13158 | orf19.5729 | CA2844 |
| IPF13229 | orf19.3876 | CA3551 |
| IPF13264 | orf19.2748 | CA1171 |
| IPF14113 | orf19.166 | CA0465 |
| IPF14255 | orf19.4767 | CA1174 |
| IPF15273 | orf19.1822 | CA0423 |
| IPF15350 | orf19.2745 | CA0215 |
| IPF16067 | orf19.3190 | CA2542 |
| IPF16368.5f | orf19.255 | CA0153 |
| IPF19614 | orf19.1496 | CA1859 |
| IPF19769 | orf19.1718 | CA2799 |
| IPF19850 | orf19.1227 | CA0208 |
| IPF19920 | orf19.4524 | CA1509 |

**Table 1.** Continued

| Candida DB | SGTC | CYGD |
|---|---|---|
| IPF20023 | orf19.6985 | CA5031 |
| IPF20024 | orf19.3012 | CA5048 |
| ARG81 | orf19.4766 | CA1175 |
| CAT8 | orf19.5097 | CA2219 |
| CTA7 | orf19.4288 | CA3060 |
| CZF1 | orf19.3127 | CA3560 |
| DAL81 | orf19.3252 | CA5449 |
| ECM22 | orf19.2623 | CA0471 |
| FCR1 | orf19.6817 | CA5890 |
| LEU3 | orf19.4225 | CA4146 |
| LYS14 | orf19.5548 | CA0404 |
| PPR1 | orf19.3986 | CA4758 |
| PUT3 | orf19.6203 | CA3214 |
| RGT1 | orf19.2747 | CA1172 |
| SEF1 | orf19.3753 | CA2346 |
| SEF11.5eoc | orf19.1926 | CA0395 |
| STB5 | orf19.3308 | CA4617 |
| SUC1 | orf19.7319 | CA5555 |

are responsible for maintaining the structure by binding two atoms of zinc (Todd and Andrianopoulos, 1997). $Cys_1$ and $Cys_4$ act by binding two zinc ions, whereas the remaining cysteine residues are terminal ligands (Figure 2) (Pan and Coleman, 1990).

The metal-binding domain is composed of two substructures with three cysteine residues in each one. $Cys_1$–$Cys_2$ and $Cys_4$–$Cys_5$ are canonically separated by two amino acid residues, while $Cys_2$–$Cys_3$ by six amino acid residues. $Cys_3$–$Cys_4$ separation is highly variable (5–16 amino acid residues) while $Cys_5$–$Cys_6$ separation has a length of 6–8 amino acid residues. The 70 OFRs found in *C. albicans* as putative $Zn(II)_2C_6$ proteins were aligned using the ClustalW program (Thompson *et al*., 1994); 25 of them exactly fit with the most restrictive pattern $CysX_2CysX_6CysX_6CysX_2CysX_6$ Cys.

Another important amino acid residue in DNA binding is a lysine residue localized between $Cys_2$ and $Cys_3$ (Figure 2). In some *S. cerevisiae* $Zn(II)_2C_6$ proteins such as Gal4p and Pdr1p (Laughon and Gesteland, 1984), this lysine residue is responsible for the specific contact with the CGG triplet in the DNA. This lysine residue is conserved in 57 of the *C. albicans* $Zn(II)_2Cys_6$ putative transcription factors (Figure 2), whereas 13 sequences contain arginine or histidine instead.

The subregion between $Cys_3$ and $Cys_4$ is highly variable. Although most of the motifs have a six

Copyright © 2006 John Wiley & Sons, Ltd. 

```
LYS14             -RVRKSYSRNGCRECKRRKIRCP-----EEKP---YCSTCVRLG--KQCSYPLAGEKVL
IPF100.3          -KKKIRRSRNGCHTCKRSKIKCD-----ENKP---TCSYCSKTK--AICDYSLKLTWGG
IPF1196           -ISKGKKSRNGCLTCKKKRLKCD-----ETKP---NCLNCTKKN--IECGGYATKFKWK
IPF928            -KFKDGRSRNGCLTCKVRKKKCS-----EEKP---VCNDCSRFG--KNCIYITDSMTEQ
IPF7289           -RKYHQKSRNGCSTCKKRRVKCD-----EQRP---VCGNCTKLK--LDCGYLHEPLENI
IPF1292           -TIKRSRTRSGCVTCRDRHIKCD-----EQQP---VCKNCQKSN--RKCYRGIRLNFTQ
IPF4835           -RIIKRRTRTGCLTCRKRRIKCD-----ERKP---TCFNCERSK--KSCLGYQDLSKLP
IPF19850          -KIKRTRSKTGCLTCRKRKKKCD-----ENKP---KCNSCIHLN--KECIWPSKDNIIS
IPF1457           -LNTSKRSRTGCLQCRARKKKCN-----EEHP---VCGSCKRRK--VNCSWRVTSKFKI
IPF1960.5F        -RFKRQRSKTGCKNCRLRKRKCD-----ELHP---TCTFCHTRD--LICEYNEIKILNP
IPF7629           -TQIKRRTKTGCLTCRKRKKKCD-----EDKVNG-KCQACTRNF--LDCCWPDPNTIKT
IPF6874.3         -RRKHNRVRTGCFTCRKRKKKCD-----EHQP---NCENCIRNK--LKCQYPSQWNEAL
ARG81             -TTRRSKTFTGCFTCRSRKIKCD-----LTKP---QCEKCTRAG--LICAGYDIKLRWS
IPF15350          -RKLGATSKTGCWTCRIRHKACP-----EEKP---SCSQCIRLQ--LDCDYSDKRPSYM
IPF15273          -RRLLPRSKKGCWICRIKHLKCD-----EVTP---ICGGCAKFG--LQCDYSSEKPAYV
IPF13264          -ARRKGRTFEGCWTCRSRKVKCD-----LTKP---QCNRCLKSN--RICQGYEIRLGWC
IPF6203           -RRKHRNSHLGCGTCKKRRIKCD-----ETLP---ACLNCLKGK--LHCAYLNLDNNAR
IPF7952           -RRRHTNSKLGCLNCKRKKVRCD-----ESLP---ECKNCVKGK-KETCSYLSLSTQEI
ECM22             -RRKHKNSKLGCANCKERRVKCL-----ENLP---SCTNCIKHR--VKCAYLDYTEDQL
CZF1              -KPITKRSRMGCLTCRQRKKRCC-----ETRP---RCTECTRLR--LNCTWPKPGTEHK
IPF8224           -IKKSKYSRGGCAECRRRKIKCD-----ELKP---YCHNCTRLN--KLCVYPTKPKFKF
IPF11777          -TSKRAYSRGGCKECKRRKIRCP-----EDKP---SCATCVRLG--KVCSYPLPGERVP
IPF13158          -SKSRSYVSIACDNCRKRRRKCN-----GELP----CHYCSGKN--KPCVYDKTKDKRR
IPF19614          -IPKRTKVSRACDLCKRHKRKCN-----GDNP----CSYCQEKS--LQCTYLKLDGRSK
IPF1034           DLDKRTKVSRACDYCKKRKFKCS-----GVSP----CELCTKKG--IQCEFNIVDRRTIRR
IPF1040           -KERRRKVSRACDYCKKRKYKCS-----GIAP----CNLCSKKQ--IDCIFSIVDKRTT
IPF10197          -LEKRTKVSRACDYCKKRKFKCS-----GVSP----CELCTKKN--IQCEFSIIDRRTI
IPF2319           -DDQRQKVSSACDNCKKRKFKCS-----GEKP----CFECSKKG--HDCTYTIIDKRSL
IPF7221           -VKVRKHVSSACLECRRRHFKCD-----GKQPV---CDRCQKSN--KPCQYVASHRGGS
RGT1              -PRKRSKVSRACDPCRKKKIKCNAEYSELEKKVTKICTSCAKNK--EICTFDRTPLKRG
IPF16067          -KKKRTRISKACEYCRKKKVKCN-----GCQP----CLNCLQSN-NGNCEYAVDDEKKP
IPF10079          -NKKRKRIRRACDFCRSKKAKCD-----GKSPV---CSNCFANK--EECIYSQPVKRRG
IPF9188           -GEKRLRTKVACDYCRKRKSKCN-----GEQP----CSKCLDKN--RNCTYTFVQKERK
IPF6510           -KRKRATNIRACDTC--KKVKCD-----ANRP----CGQCRTNN--LECTHTRERKKTG
IPF14113          -ENKRRRVTRACDTCRQKKVKCD-----GKQP----CIHCTVYS--YKCSYDQPNIRNK
IPF9312           -TYPRKRALTACDTCRLKKIKCD-----NVRPR---CGSCIKNG-NLNCHYRTDDQQKD
FCR1              -HIKKKRVGKACDSCRIKKTKCD-----GKKP----CNRCTLDN--KICVFTEKKKTKE
IPF776            -KQPPYPIEQACDSCRKRKLKCS-----KEFP---RCSKCIQHE--WCCSYSPRTIRS
IPF9499           -TSSGVRVSQACDRCRIKKIKCD-----GQSP----CRNCQKVS--VECKTS-DRLSRKS
CAT8              -GSKVERVAQACDRCRAKKTKCD-----GQNP----CSTCQSVG--LECIVS-DRLTRKS
CTA7              -SKQRMRVVKACDRCRSHKIKCS-----GECP----CATCLKQA--KECTFS-NKVFQKR
IPF13229          -YAQASRTLKACELCRKQKTRCFRS---PENPNS--CLRCRFLS--KTCSFENEETEGE
IPF14255          -KKYSPRSRHACNNCRLKKLKCD-----EQKPQ---CSRCSKKGYD--CVYHFNVQFKQ
PPR1              -SSKLTRAISACKRCRTKKIKCD-----QKFPQ---CGKCEVNG--VECIGV-DSVTGRE
IPF6554           -KKNRKPAVRACVFCHQKHLQCS-----NERP----CKNCVKRNIAHGCQDIVRKRVKY
IPF20024          -QGKYKRNYRACLNCRLRKVKCDLGP--VDNPHDGKCARCLRER--KDCVFVESKRGGT
IPF907            -FSKQWRQSRACTRCRRFKKKCS-----FENPSFKSCARCFKNG--YECSFNEDPAMQP
LEU3              -SKTKRSKKMACVECRQQKSRCDAF---EKRPDP--CTRCAKKGLH--CDVKSDYKRTY
CWT1              -KRRKKKLEIACVYCRRSHMICD-----ESRP----CQRCIKRGIAHLCYDEPSNSRQR
PUT3              -NPRQKRASLACIRCRTRHIRCP-----GGDP----CRKCQLAK--SKCEYVEADKKIV
IPF6159           -KPKVTRRSVACKSCHSLKVKCTPSDPNNPSAP---CVRCINAN--RICEIDLNQTRKR
IPF376            -STTTPRASVACNLCRSSKIKCIN---NSDSTR---CHRCASLD--LECTYTLKTSQLK
IPF9251           -GQKRNRISKVCDFCKRRKVKCD-----LGNP----CSMCVKYK-KSPCVYSEQPTEAP
IPF1264           -VKKRNRATLVCLSCRKKKVKCD-----KGKP----CSNCNKSQ-PDKCIYDERISESK
IPF1266           -VRKRKKVSTVCTNCRKRKIRCD-----RQHP----CNNCIKSKKHNACVYDDGQVSPA
IPF13021          -TKKRNRLSLNCFNCKRKKIKCN-----RQQP----CLSCIKSNLESKCEYQQQKWLAT
SEF11.5eoc        -PPKGGRILKTCTRCRRHKTKCDAQ---DTNPYP--CSHCFKRN--LDCTLETISKTGG
IPF20023          -APPRTEKRISCQRCRTRKIKCN-----YELP----CFNCKRDG--SQCIQPIDMRSKR
IPF3444.3f        -MTKRDRTIYSCDACRSRKIKCN-----RQTP----CASCHKSK--RDCVYTVSRQRDA
IPF19769          -KPKRQRRSYSCGPCKLLKIKCD-----LQIP----CTACKKFNRVNRCLLQPPQPPSQ
IPF10533.exon1    -QKTRQRRILSCVYCHSKKIKCD-----RQKP----CSQCTKLGME--CKYFINERISR
STB5              -HPEDMPKLTSCLRCRKLKKKCD-----KSTP---HCLNCENAN--EECTYVKRKPRQS
SEF1              -KPTGHRPVTSCTFCRQHKIKCNAS--DNYPNP---CERCKKMG--LKCEIDPEFRPRK
IPF9826           -KRQRNRVPVSCLACKKRKVKCD-----KGKPA---CGGCVRNGVGHLCQYINPPWIDN
IPF16368.5f       -MSSALLP-ISCISCRKRKIKC------NRIKP---CDQCIKRH--LPCEFPEKFRNIK
IPF13024          -KRSRVRQPLSCSVCRKRKSKCD-----RARP----CGTCIKKSIVHLCHYEDDNRPPI
SUC1              -KGKRAPYTRPCDSCSFRKVKCD-----MKTP----CSRCVLNN--LKCTNNRIRKKCG
DAL81             -SSKSNKQKRPCDQCRKRKIKCV------LVPNTNNCVQCEAKQ--VTCTYTDQAPKRK
IPF2029           --DKKEKRTKPCCNCKRSKVKCVYT---SSLP----CERCVKTG--QTCQFVPKLPSFK
IPF19920.3        -------MNYRCNRCRSKKTKCD-----GGFP----CLKCVKSN--QECNISAPERPSN
```

**Figure 2.** Alignment of the $C_6$ zinc cluster region in *C. albicans* proteins. The colour code is as follows: Cys, yellow and highlighted; Arg, His and Lys, blue (residues making specific DNA contact are also highlighted and in italics); Met, Val, Leu, Ala and Ile, grey; Glu, Asp, pink; Phe, Tyr and Trp, purple; Gln, Ser, Asn and Thr, green; Gly and Pro, red amino acid residues extension, its variability ranges from five to 16 residues. A proline residue is present in almost all the ORFs identified (Figure 2), maybe involved in the turn required between the two $\alpha$-helix subregions. The subregions between $Cys_2$ and $Cys_3$ and between $Cys_5$ and $Cys_6$ are rich in basic amino acids. A consensus sequence in the N- and C-terminal regions flanking the six cysteines domain has not been found. However, there is a predominance of basic amino acid residues (Lys and Arg) at both the N-terminus and at the C-terminus, but to a lesser extent. These basic domains could permit or enhance the DNA recognition.

### Structure of the *C. albicans* $Zn(II)_2C_6$ proteins

$Zn(II)_2C_6$ transcription regulator factors are composed of two clearly different domains responsible for DNA binding and activation (Todd and Andrianopoulos, 1997, and references herein). In *C. albicans*, the $Zn(II)_2C_6$ domain is usually found at the N-terminal region of the protein with a few exceptions: five at the C-terminus and one in the middle of the protein (Figure 3).

Whiteway *et al.* (1992) identified the gene *CFZ1* that conferred moderate pheromone resistance on *S. cerevisiae*. Czf1p shows an overall structure that resembles that of a transcription factor, with a glutamine-rich region in the central part and a cysteine-rich region at the C-terminus of the protein.

The similarity outside the zinc cluster region reported for some *S. cerevisiae* $Zn(II)_2C_6$ proteins has also been found in *C. albicans*. It has been discovered that some putative fungal proteins contain a characteristic domain involved in transcription control (**http://www.sanger.ac.uk/cgi-bin/Pfam/getacc?PF04082**), although their function has not been elucidated to date (Marczak and Brandriss, 1991; Hedges *et al.*, 1995; Kasten and Stillman, 1997; van Peij *et al.*, 1998). The search in the *C. albicans* database for $Zn(II)_2C_6$ proteins containing such fungal domain revealed the occurrence of at least 12 ORFs (Figure 3). Moreover, some of these ORFs (PUT3, STB5, CAT8, PPR1, IPF20023 and DAL81) present a high level of identity with their respective *S. cerevisiae* homologues.

SCANPROSITE analysis (Gattiker *et al.*, 2002) revealed the presence of other interesting motifs (Figure 3): (a) glutamine-rich regions, which may form hydrogen bonds with target factors (Courey and Tjian, 1988), resembling those previously described for other transcription factors (Aro *et al.*, 2001); (b) proline-rich regions which may fold into a unique structure that forms protein–protein contact with the transcription machinery (Mermod *et al.*, 1989) — IPF10079 and IPF9499 present such region at the C-terminus and RGT1 and IPF13024 at the N-terminus of the protein, close to the $Zn(II)_2C_6$ motif; (c) histidine, serine and threonine-rich regions (Figure 2); (d) basic leucine zipper domains, frequent in both *S. cerevisiae* (Fernandes *et al.*, 1997) and *C. albicans* (Yang *et al.*, 2001). These motifs are implicated in protein dimerization (Busch and Sassone-Corsi, 1990).

The global analysis of these proteins using the PSORTII program (Horton and Nakai, 1997) exhibits the presence of a nuclear localization signal (Talibi and Raymond, 1999; Moreno *et al.*, 2003) in most of them, suggesting a putative nuclear localization (Table 2).

The presence of coiled-coil elements was searched for the 70 sequences by the COIL program (Taylor and Zhulin, 1999), to investigate the potential of dimerization via this structure. The program described by Lupas *et al.* (1991) assigns a score to each amino acid residue included in a window with 7, 14 or 28 residues (two, three or four heptads) on the basis of their probability of being involved in a coiled-coil structure. Positive scores were only reported when probability values were $>0.9$ in the 150 residues of the C-terminal $Zn(II)_2C_6$ domain (Table 2). From the 65 putative proteins with an N-terminal $Zn(II)_2C_6$ domain, a high peak was detected in 28, 21 and 17 for a two-, three- or four-heptad window, respectively. This analysis shows that the occurrence of a coiled-coil region situated at the C-terminus of the $Zn(II)_2C_6$ domain is quite frequent in these putative transcription factors, and is probably involved in dimerization events.

A transcription factor (CaCwt1p), required for cell wall integrity, has been recently characterized by our group (Moreno *et al.*, 2003). CaCwt1p has been structurally analysed and the presence of another family of C-terminal motifs has been predicted. This region, named PAS, is presumed to be involved in eukaryotic signal transduction or dimerization events (Taylor and Zhulin, 1999). The

Copyright © 2006 John Wiley & Sons, Ltd. 

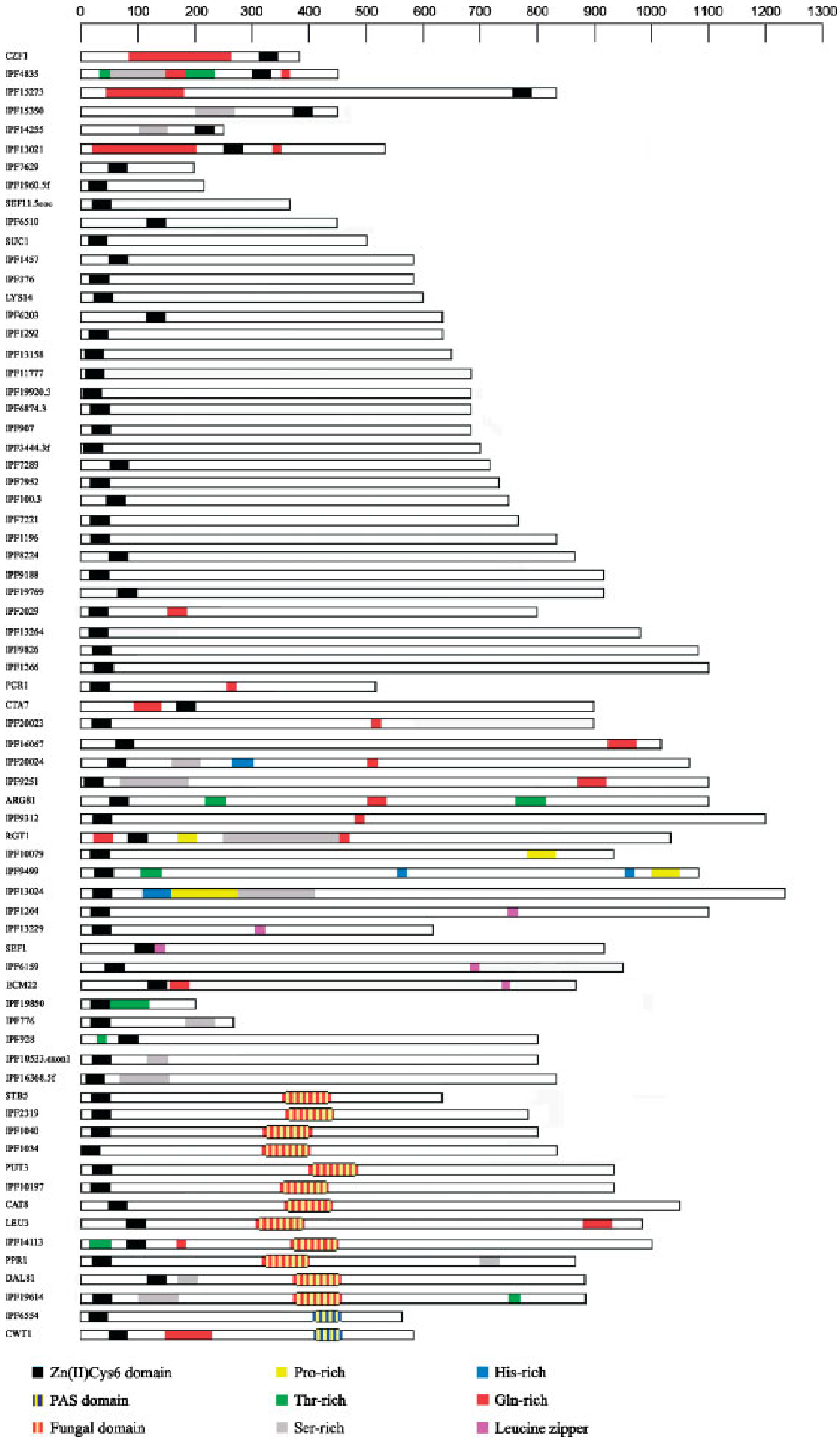

**Figure 3.** Schematic structural representation of the most common structural features predicted for the *C. albicans* $Zn(II)_2Cys_6$ protein family

**Table 2.** Summary of data for the *Candida albicans* $Zn(II)_2Cys_6$ cluster proteins

| ORF name | Nuclear localization (%) | Last Cys | Coiled-coil probability >0.9 (within 150 residues) | | |
|---|---|---|---|---|---|
| | | | Two heptads | Three heptads | Four heptads |
| IPF16368.5f | 94.1 | 36 | + | + | + |
| IPF19850 | 94.1 | 45 | − | − | − |
| IPF15350 | 94.1 | Ct | na | na | na |
| IPF10079 | 94.1 | 45 | − | − | − |
| SEF11.5eoc | 56.5 | 59 | − | − | + |
| LYS14 | 4.3 | 67 | − | − | − |
| IPF15273 | 87.0 | Ct | na | na | na |
| IPF14113 | 69.6 | 61 | − | − | − |
| ECM22 | 82.6 | 143 | + | + | + |
| IPF11777 | 95.7 | 40 | − | − | − |
| IPF6159 | 73.9 | 86 | + | + | + |
| IPF1034 | 94.1 | 42 | − | − | − |
| IPF13264 | 31.8 | 44 | + | + | − |
| RGT1 | 69.6 | 125 | − | − | − |
| IPF14255 | 78.3 | Ct | na | na | na |
| ARG81 | 52.2 | 135 | + | − | − |
| IPF19920.3 | 13.0 | 31 | − | − | − |
| IPF1040 | 100.0 | 43 | − | − | − |
| IPF9312 | 21.7 | 267 | − | − | − |
| IPF13021 | 65.2 | 121 | − | − | − |
| IPF7629 | 87.0 | 84 | − | − | − |
| IPF19614 | 43.5 | 57 | − | − | − |
| IPF10197 | 21.7 | 41 | + | − | − |
| IPF13024 | 82.6 | 61 | − | − | − |
| IPF6874.3 | 21.7 | 51 | − | − | − |
| CAT8 | 65.2 | 80 | + | + | + |
| IPF8224 | 65.2 | 71 | − | − | − |
| IPF6510 | 65.2 | 141 | − | − | − |
| SEF1 | 73.9 | 120 | + | + | + |
| IPF9188 | 52.2 | 52 | + | + | + |
| IPF16067 | 43.5 | 93 | + | + | + |
| IPF9499 | 69.6 | 58 | + | + | + |
| IPF19769 | 21.7 | 97 | + | + | + |
| IPF13158 | 26.1 | 39 | − | − | − |
| CWT1 | 78.3 | 79 | − | − | − |
| CTA7 | 73.9 | 201 | − | − | − |
| IPF9826 | 43.5 | 67 | + | + | + |
| IPF3444.3f | 0.0 | 37 | + | − | − |
| PUT3 | 69.6 | 57 | + | + | + |
| IPF10533.exon1 | 65.2 | 74 | + | + | + |
| IPF13229 | 65.2 | 74 | − | − | − |
| CZF1 | 73.9 | Ct | na | na | na |
| IPF9251 | 39.1 | 39 | + | + | − |
| IPF6203 | 82.6 | 155 | − | − | − |
| IPF7289 | 65.2 | 141 | − | − | − |
| IPF7221 | 73.9 | 48 | − | − | − |
| LEU3 | 65.2 | 112 | + | + | − |
| IPF2319 | 17.4 | 57 | + | + | + |
| IPF7952 | 43.5 | 46 | − | − | − |
| STB5 | 22.2 | 66 | − | − | − |
| IPF6554 | 95.7 | 48 | − | − | − |
| PPR1 | 69.6 | 62 | + | − | − |
| IPF1196 | 69.6 | 55 | − | − | − |

Copyright © 2006 John Wiley & Sons, Ltd. 

**Table 2.** Continued

| ORF name | Nuclear localization (%) | Last Cys | Coiled-coil probability >0.9 (within 150 residues) | | |
|---|---|---|---|---|---|
| | | | Two heptads | Three heptads | Four heptads |
| IPF1457 | 87.0 | 78 | − | − | − |
| IPF2029 | 69.6 | 45 | + | + | − |
| IPF20023 | 43.5 | 68 | + | − | + |
| IPF20024 | 60.9 | 78 | − | − | − |
| DAL81 | 65.2 | 149 | − | − | − |
| IPF776 | 69.6 | 50 | + | − | − |
| IPF1960.5f | 65.2 | 44 | − | − | − |
| SUC1 | 47.8 | 39 | − | − | − |
| IPF1264 | 69.6 | 50 | + | + | + |
| IPF1266 | 47.8 | 59 | + | + | + |
| IPF1292 | 65.2 | 46 | − | − | − |
| IPF376 | 11.1 | 43 | − | − | − |
| FCR1 | 82.6 | 52 | + | + | − |
| IPF928 | 21.7 | 101 | + | + | − |
| IPF907 | 39.1 | 55 | + | − | − |
| IPF4835 | 56.5 | Ct | na | na | na |
| IPF100.3 | 21.7 | 70 | − | − | − |

Ct, located at the C-terminus of the protein.
Nuclear localization was predicted with PSORT-II (Horton and Nakai, 1997). Coiled-coil probability within the C-terminal region of the Zn(II)$_2$Cys$_6$ domain was calculated by COILS (Lupas *et al.*, 1991).

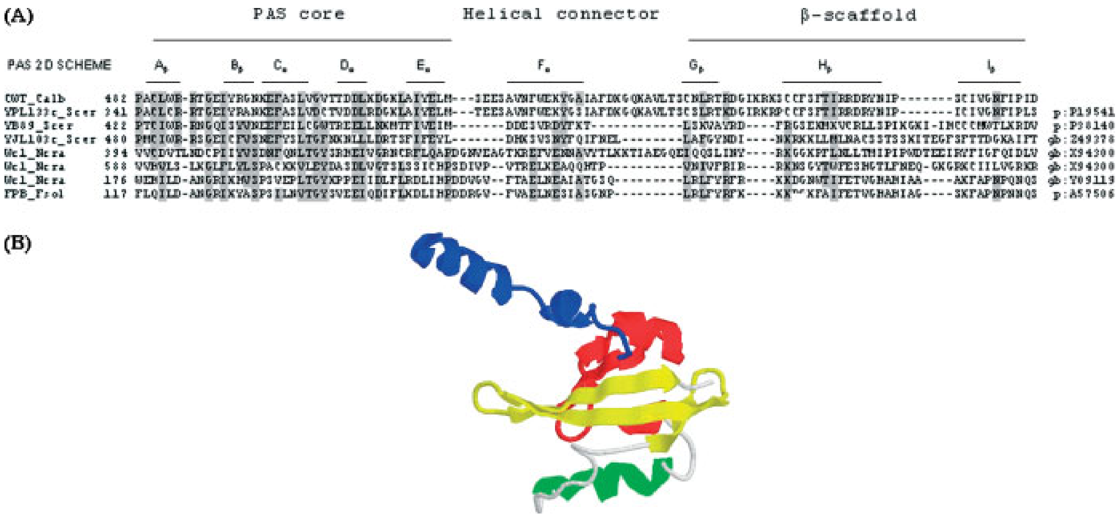

**Figure 4.** (A) Multiple structure-based alignment of fungal PAS domains. The alignment was performed as described by Taylor and Zhulin (1999), with modifications. (B) The secondary structure of CaCwt1p was deduced from that of *Rhizobium melitoti* FixL (PDB: 1ew0). Similar residues in at least four sequences are highlighted. The N-terminal link region (blue), PAS core (red), helical connector (green) and $\beta$-scaffold (yellow) are shown. The figure was generated using RasMol (Sayle and Milner-White 1995)

putative protein IPF6554 also possesses such characteristic domain. A multiple structure-based alignment also including the *S. cerevisiae* sequences for CaCwt1p, YPL133c and YJL103c, respectively, is shown in Figure 4. The biological function of the PAS domain in yeast proteins has not been elucidated yet. Although many of the transcription factors described at present can be basically depicted as three-component proteins, where the DNA recognition motif is linked to a dimerization region by a function variable spacer, this is not a general rule. This general structure is not always

Copyright © 2006 John Wiley & Sons, Ltd. 

**Table 3.** Comparison between genes encoding putative zinc cluster proteins in *S. cerevisiae* and *C. albicans*. Only positive results from reciprocal BLAST are included

| *Saccharomyces cerevisiae* | | | | *Candida albicans* | |
|---|---|---|---|---|---|
| **Systematic name** | **Gene** | **Function** | **Ref.** | **Systematic name** | **Ref.** |
| YDR207c | UME6 | Regulator of early meiotic genes | a | IPF15350 | — |
| YDR034c | LYS14 | Transcriptional activator of lysine pathway genes | b | LYS14 | — |
| YIL130w | — | Unknown | — | IPF14113 | — |
| YML076c | WAR1 | Regulate weak acid stress response | c | IPF6159 | — |
| YKL038w | RGT1 | Constitutive expression of glucose-induced HXT genes | d | RGT1 | — |
| YML099c | ARG81 | Regulator of arginine responsive genes | e | ARG81 | — |
| YBL066c | SEF1 | Suppressor of essential function | e | SEF1 | — |
| YPL133c | RDS2 | Unknown | e | CWT1 | i |
| YMR019c | STB4 | Unknown | f | CTA7 | — |
| YLR256c | HAP1 | Regulator of oxygen-dependent genes | e | IPF9826 | — |
| YKL015w | PUT3 | Regulator of Pro utilization genes | g | PUT3 | — |
| YDR520c | — | Unknown | — | IPF13229 | — |
| YOR363c | PIP2 | Activator of peroxisome proliferation | e | IPF9251 | — |
| YDR213w | UPC2 | Involved in sterol uptake | e | IPF7289 | — |
| YLR451w | LEU3 | Regulator of amino acid biosynthesis | e | LEU3 | — |
| YHR178w | STB5 | Binds Sin3p in two-hybrid assay | f | STB5 | — |
| YJL103c | — | Unknown | h | IPF6554 | — |
| YLR014c | PPR1 | Activator of URA1 and URA3 genes | h | PPR1 | — |
| YOR337w | TEA1 | Activator of Ty1 elements | e | IPF20023 | — |
| YDR421w | ARO80 | Unknown | e | IPF20024 | — |
| YIR023w | DAL8 | Regulator of nitrogen catabolic genes | e | DAL81 | — |
| YFL052w | — | Unknown | e | SUC1 | — |
| YDL170w | UGA3 | Unknown | h | IPF928 | — |

References: a, Tong *et al.* 2004; b, El Alami *et al.* 2002; c, Kren *et al.* 2003; d, Kim *et al.* 2003; e, Akache *et al.* 2001; f, Kasten & Stillman 1997; g, Axelrod *et al.* 1991; h, Giaever *et al.* 2002: i, Moreno *et al.* 2003.

present and some well-characterized proteins lack this dimerization motif (Anderson *et al.*, 1995).

The function of most of the *C. albicans* $Zn(II)_2C_6$ proteins remains uncharacterized, although a few cluster proteins, involved in several biological functions, have been reported. CaSuc1p is a transcription factor involved in sucrose utilization by affecting an inducible $\alpha$-glucosidase, and was the first $Zn(II)_2C_6$ zinc finger protein described in *C. albicans* (Kelly and Kwon-Chung, 1992). The *CaCFR1* gene encoding a $Zn(II)_2C_6$ protein was isolated by its ability to complement the fluconazole hypersensitivity of a *S. cerevisiae* mutant lacking the transcription factors Pdr1p and Pdr3p (Talibi and Raymond, 1999). An atypical protein with a C-terminal $Zn(II)_2C_6$ motif, CaAzf1p, has also been reported previously (Whiteway *et al.*, 1992).

Some other *C. albicans* putative $Zn(II)_2C_6$ transcription regulators have also been tentatively assigned by comparison with their corresponding *S. cerevisiae* homologues (Table 3). However, the functional role of these similarities remains unclear, and could be related to evolutionary aspects. As an example, some zinc cluster proteins control the expression of genes required for gluconeogenesis in *S. cerevisiae*, such as Cat8p (Hedges *et al.*, 1995), Arg81p (Messenguy, 1976), Lys14p (Ramos *et al.*, 1988) and Ppr1p (Marmorstein and Harrison, 1994), involved in the metabolism of arginine, lysine and pyrimidines, respectively, which have homologues in *C. albicans* with a high sequence identity. Another member of the $Zn(II)_2C_6$ protein family, CaPut3p, involved in controlling enzymes required for proline use as a nitrogen source, was previously characterized in *S. cerevisiae* (Marczak and Brandriss, 1989).

### Evolutionary relationships among $Zn(II)_2C_6$ clusters

Phylogenetic analysis was performed using the entire DNA binding region, including the $Zn(II)_2C_6$

Copyright © 2006 John Wiley & Sons, Ltd. 

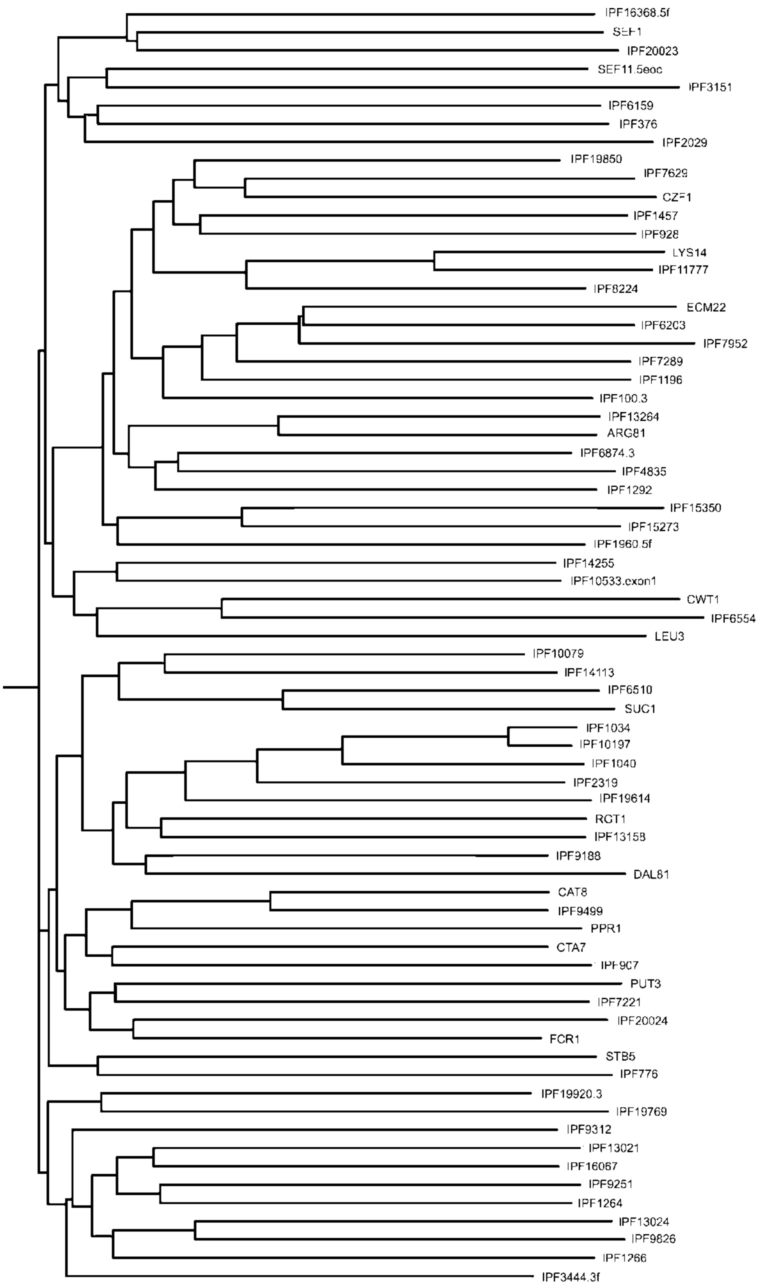

**Figure 5.** Phenogram of Zn(II)2Cys6 domains. Proteins containing $Zn(II)_2Cys_6$ domains have been obtained from the *C. albicans* genome database at **http://www-sequence.stanford.edu/group/candida**. The 10 N- and C-terminal flanking nucleotides were included within the input sequences cluster and N- and C-terminal flanking sequences of the 70 predicted *C. albicans* proteins. After alignment using the ClustalW program (Figure 2), the output data were submitted to the Phylip drawtree web interface utility and a phenogram was obtained (Figure 5).

Sometimes there is strong support for grouping as inferred from bootstrap analysis. All the $Zn(II)_2C_6$ proteins containing the fungal domain previously described, with the single exception of LEU3, have been clustered in a single branch. This consistent association of unknown proteins

Copyright © 2006 John Wiley & Sons, Ltd.

could represent regulation via a common pathway, although this remains to be elucidated. CWT1 and IPF6554 have also been consistently clustered on the basis of their $Zn(II)_2C_6$ domains. This data, together with the presence of the PAS domain in both these two putative proteins suggests their possible involvement in similar functional processes that should be investigated.

### Acknowledgements

This work was partially supported by grants from the Ministerio de Ciencia y Tecnología, Spain (BMC2000-1449), the Program Quality of Life and Management of Living Resources, Brussels (QLK2-CT-2000-00795) and the Agència Valenciana de Ciència i Tecnologia de la Generalitat Valenciana, Spain (G03/187).

### References

Akache B, Wu K, Turcotte B. 2001. Phenotypic analysis of genes encoding yeast zinc cluster proteins. *Nucleic Acids Res* **29**: 2181–2190.

Altschul SF, Madden TL, Schaffer AA, *et al*. 1997. Gapped BLAST and PSI-BLAST: a new generation of protein database search programs. *Nucleic Acids Res* **25**: 3389–3402.

Anderson SF, Steber CM, Esposito RE, Coleman JE. 1995. *UME6*, a negative regulator of meiosis in *Saccharomyces cerevisiae*, contains a C-terminal Zn2Cys6 binuclear cluster that binds the URS1 DNA sequence in a zinc-dependent manner. *Protein Sci* **4**: 1832–1843.

Aro N, Saloheimo A, Ilmen M, Penttila M. 2001. ACEII, a novel transcription activator involved in regulation of cellulase and xylanase genes of *Trichoderma reesei*. *J Biol Chem* **276**: 24 309–24 314.

Ascone I, Lenouvel F, Sequeval D, Dexpert H, Felenbok B. 1997. First experimental evidence of a zinc binuclear cluster in AlcR protein, mutational and X-ray absorption studies. *Biochim Biophys Acta* **1343**: 211–220.

Axelrod JD, Majors J, Brandriss MC. 1991. Proline-independent binding of *PUT3* transcription activator protein detected by footprinting *in vivo*. *Mol Cell Biol* **11**: 564–567.

Bates PA, Sternberg MJ. 1999. Model building by comparison at CASP3: using expert knowledge and computer automation. *Proteins* Suppl **3**: 47–54.

Breunig KD, Kuger P. 1987. Functional homology between the yeast regulatory proteins Gal4 and Lac9: Lac9-mediated transcription activation in *Kluyveromyces lactis* involves protein binding to a regulatory sequence homologous to the Gal4 protein-binding site. *Mol Cell Biol* **7**: 4400–4406.

Burglin TR. 1991. The TEA domain: a novel, highly conserved DNA-binding motif. *Cell* **12**: 11–12.

Busch SJ, Sassone-Corsi P. 1990. Dimers, leucine zippers and DNA-binding domains. *Trends Genet* **6**: 36–40.

Courey AJ, Tjian R. 1988. Analysis of Sp1 *in vivo* reveals multiple transcription domains, including a novel glutamine-rich activation motif. *Cell* **55**: 887–898.

Davis MA, Hynes MJ. 1987. Complementation of *areA*-regulatory gene mutations of *Aspergillus nidulans* by the heterologous regulatory gene *nit*-2 of *Neurospora crassa*. *Proc Natl Acad Sci USA* **84**: 3753–3757.

El Alami M, Feller A, Pierard A, Dubois E. 2002. The proper folding of a long C-terminal segment of the yeast Lys14p regulator is required for activation of *LYS* genes in response to the metabolic effector. *Mol Microbiol* **43**: 1629–1639.

Fernandes L, Rodrigues-Pousada C, Struhl K. 1997. Yap, a novel family of eight bZIP proteins in *Saccharomyces cerevisiae* with distinct biological functions. *Mol Cell Biol* **17**: 6982–6993.

Furst P, Hu S, Hackett R, Hamer D. 1988. Copper activates metallothionein gene transcription by altering the conformation of a specific DNA binding protein. *Cell* **55**: 705–717. Erratum in: *Cell* 1989 Jan 27; 56(2): 321 ff.

Gattiker A, Gasteiger E, Bairoch A. 2002. ScanProsite: a reference implementation of a PROSITE scanning tool. *Appl Bioinformat* **1**: 107–108.

Giaever G, Chu AM, Ni L, *et al*. 2002. Functional profiling of the *Saccharomyces cerevisiae* genome. *Nature* **418**: 387–391.

Guex N, Peitsch MC. 1997. SWISS-MODEL and the Swiss-PdbViewer: an environment for comparative protein modeling. *Electrophoresis* **18**: 2714–2723.

Hedges D, Proft M, Entian KD. 1995. CAT8, a new zinc cluster-encoding gene necessary for derepression of gluconeogenic enzymes in the yeast *Saccharomyces cerevisiae*. *Mol Cell Biol* **15**: 1915–1922.

Horton P, Nakai K. 1997. Better prediction of protein cellular localization sites with the *k* nearest neighbours classifier. *Proc Int Conf Intell Syst Mol Biol* **5**: 147–152.

Jakobsen BK, Pelham HRB. 1991. A conserved heptapeptide restrains the activity of the yeast heat shock transcription factor. *EMBO J* **10**: 369–375.

Kammerer B, Guyonvarch A, Hubert JC. 1984. Yeast regulatory gene *PPR1*. I. Nucleotide sequence, restriction map and codon usage. *J Mol Biol* **180**: 239–250.

Kasten MM, Stillman DJ. 1997. Identification of the *Saccharomyces cerevisiae* genes *STB1–STB5* encoding Sin3p binding proteins. *Mol Gen Genet* **256**: 376–386.

Kelly R, Kwon-Chung KJ. 1992. A zinc finger protein from *Candida albicans* is involved in sucrose utilization. *J Bacteriol* **174**: 222–232.

Kim JH, Polish J, Johnston M. 2003. Specificity and regulation of DNA binding by the yeast glucose transporter gene repressor Rgt1. *Mol Cell Biol* **23**: 5208–5216.

Klug A, Rhodes D. 1987. 'Zinc fingers': a novel protein motif for nucleic acid recognition. *Trends Biochem Sci* **12**: 464–469.

Kohler T, Wesche S, Taheri N, Braus GH, Mosch HU. 2002. Dual role of the *Saccharomyces cerevisiae* TEA/ATTS family transcription factor Tec1p in regulation of gene expression and cellular development. *Eukaryot Cell* **1**: 673–686.

Kren A, Mamnun YM, Bauer BE, *et al*. 2003. War1p, a novel transcription factor controlling weak acid stress response in yeast. *Mol Cell Biol* **23**: 1775–1785.

Laughon A, Gesteland RF. 1984. Primary structure of the *Saccharomyces cerevisiae GAL4* gene. *Mol Cell Biol* **4**: 260–267.

Li D, Kolattukudy PE. 1997. Cloning of cutinase transcription factor 1, a transactivating protein containing Cys6Zn2 binuclear cluster DNA-binding motif. *J Biol Chem* **272**: 12 462–12 467.

Copyright © 2006 John Wiley & Sons, Ltd. 

Lim A, Zhang L. 1999. WebPHYLIP: a web interface to PHYLIP. *Bioinformatics* **15**: 1068–1069.

Lupas A, Van Dyke M, Stock J. 1991. Predicting coiled coils from protein sequences. *Science* **252**: 1162–1164.

Marczak JE, Brandriss MC. 1989. Isolation of constitutive mutations affecting the proline utilization pathway in *Saccharomyces cerevisiae* and molecular analysis of the *PUT3* transcription activator. *Mol Cell Biol* **9**: 4696–4705.

Marczak JE, Brandriss MC. 1991. Analysis of constitutive and noninducible mutations of the *PUT3* transcription activator. *Mol Cell Biol* **11**: 2609–2619.

Marmorstein R, Harrison SC. 1994. Crystal structure of a PPR1–DNA complex: DNA recognition by proteins containing a Zn2Cys6 binuclear cluster. *Genes Dev* **8**: 2504–2512.

Mermod N, O'Neill EA, Kelly TJ, Tijan R. 1989. The proline-rich transcription activator of CTF/NF-I is distinct from the replication and DNA binding domain. *Cell* **58**: 741–753.

Messenguy F. 1976. Regulation of arginine biosynthesis in *Saccharomyces cerevisiae*: isolation of a *cis*-dominant, constitutive mutant for ornithine carbamoyltransferase synthesis. *J Bacteriol* **128**: 49–55.

Moreno I, Pedreño Y, Maicas S, *et al*. 2003. Characterization of a *Candida albicans* gene encoding a putative transcription factor required for cell wall integrity. *FEMS Microbiol Lett* **226**: 159–167.

Nehlin JO, Ronne H. 1990. Yeast *MIG1* repressor is related to the mammalian early growth response and Wilms' tumour finger proteins. *EMBO J* **9**: 2891–2898.

Pan T, Coleman JE. 1990. GAL4 transcription factor is not a 'zinc finger' but forms a Zn(II)2Cys6 binuclear cluster. *Proc Natl Acad Sci USA* **87**: 2077–2081.

Ramos F, Dubois E, Pierard A. 1988. Control of enzyme synthesis in the lysine biosynthetic pathway of *Saccharomyces cerevisiae*. Evidence for a regulatory role of gene *LYS14*. *Eur J Biochem* **15**: 171–176.

Talibi D, Raymond M. 1999. Isolation of a putative *Candida albicans* transcription regulator involved in pleiotropic drug resistance by functional complementation of a *pdr1 pdr3* mutation in *Saccharomyces cerevisiae*. *J Bacteriol* **181**: 231–240.

Tang CS, Bueno A, Russell P. 1994. *ntf1* + encodes a 6-cysteine zinc finger-containing transcription factor that regulates the *nmt1* promoter in fission yeast. *J Biol Chem* **269**: 11 921–11 926.

Taylor BL, Zhulin IB. 1999. PAS domains: internal sensors of oxygen, redox potential, and light. *Microbiol Mol Biol Rev* **63**: 479–506.

Thompson J, Higgins DG, Gibson TJ. 1994. ClustalW: improving the sensitivity of progressive multiple sequence alignment through sequence weighting, position-specific gap penalties and weight matrix choice. *Nucleic Acids Res* **22**: 4673–4680.

Todd RB, Andrianopoulos A. 1997. Evolution of a fungal regulatory gene family: the $Zn(II)_2Cys_6$ binuclear cluster DNA binding motif. *Fungal Genet Biol* **21**: 388–405.

Todd RB, Murphy RL, Martin HM, *et al*. 1997. The acetate regulatory gene *facB* of *Aspergillus nidulans* encodes a Zn(II)2Cys6 transcription activator. *Mol Gen Genet* **254**: 495–504.

Tong AH, Lesage G, Bader GD, *et al*. 2004. Global mapping of the yeast genetic interaction network. *Science* **303**: 808–813.

Vallee BL, Coleman JE, Auld DS. 1991. Zinc fingers, zinc clusters, and zinc twists in DNA-binding protein domains. *Proc Natl Acad Sci USA* **88**: 999–1003.

Van Peij NN, Visser J, De Graaff LH. 1998. Isolation and analysis of XlnR, encoding a transcription activator co-ordinating xylanolytic expression in *Aspergillus niger*. *Mol Microbiol* **27**: 131–142.

Whiteway M, Dignard D, Thomas DY. 1992. Dominant negative selection of heterologous genes: isolation of *Candida albicans* genes that interfere with *Saccharomyces cerevisiae* mating factor-induced cell cycle arrest. *Proc Natl Acad Sci USA* **89**: 9410–9414.

Woloshuk CP, Foutz KR, Brewer JF, *et al*. 1994. Molecular characterization of *aflR*, a regulatory locus for aflatoxin biosynthesis. *Appl Environ Microbiol* **60**: 2408–2414.

Yang X, Talibi D, Weber S, Poisson G, Raymond M. 2001. Functional isolation of the *Candida albicans FCR3* gene encoding a bZip transcription factor homologous to *Saccharomyces cerevisiae* Yap3p. *Yeast* **18**: 1217–1225.

Yuan GF, Fu YH, Marzluf GA. 1991. *nit-4*, a pathway-specific regulatory gene of *Neurospora crassa*, encodes a protein with a putative binuclear zinc DNA-binding domain. *Mol Cell Biol* **11**: 5735–5745.

Copyright © 2006 John Wiley & Sons, Ltd. 
